# Intestinal microbiota composition is altered according to nutritional biorhythms in the leopard coral grouper (*Plectropomus leopardus*)

**DOI:** 10.1371/journal.pone.0197256

**Published:** 2018-06-01

**Authors:** Miyuki Mekuchi, Taiga Asakura, Kenji Sakata, Tomofumi Yamaguchi, Kazuhisa Teruya, Jun Kikuchi

**Affiliations:** 1 RIKEN Center for Sustainable Resource Science, Tsurumi-ku, Yokohama, Kanagawa, Japan; 2 National Fishery Research Institute of Fishery Sciences, Fishery Research and Education Organization, Kanazawa-ku, Yokohama, Japan; 3 Research Center for Subtropical Fisheries, Ishikagi, Japan; 4 Graduate School of Medical Life Science, Yokohama City University, Tsurumi-ku, Yokohama, Kanagawa, Japan; 5 Graduate School of Bioagricultural Sciences, Nagoya University, Chikusa-ku, Nagoya, Aichi, Japan; Texas A&M University, UNITED STATES

## Abstract

Aquaculture is currently a major source of fish and has the potential to become a major source of protein in the future. These demands require efficient aquaculture. The intestinal microbiota plays an integral role that benefits the host, providing nutrition and modulating the immune system. Although our understanding of microbiota in fish gut has increased, comprehensive studies examining fish microbiota and host metabolism remain limited. Here, we investigated the microbiota and host metabolism in the coral leopard grouper, which is traded in Asian markets as a superior fish and has begun to be produced via aquaculture. We initially examined the structural changes of the gut microbiota using next-generation sequencing and found that the composition of microbiota changed between fasting and feeding conditions. The dominant phyla were *Proteobacteria* in fasting and *Firmicutes* in feeding; interchanging the dominant bacteria required 12 hours. Moreover, microbiota diversity was higher under feeding conditions than under fasting conditions. Multivariate analysis revealed that *Proteobacteria* are the key bacteria in fasting and *Firmicutes* and *Fusobacteria* are the key bacteria in feeding. Subsequently, we estimated microbiota functional capacity. Microbiota functional structure was relatively stable throughout the experiment; however, individual function activity changed according to feeding conditions. Taken together, these findings indicate that the gut microbiota could be a key factor to understanding fish feeding conditions and play a role in interactions with host metabolism. In addition, the composition of microbiota in ambient seawater directly affects the fish; therefore, it is important to monitor the microbiota in rearing tanks and seawater circulating systems.

## Introduction

Marine products are one of the main sources of protein and other valuable nutrients [1–3]. With the increasing demand for marine products, aquaculture has expanded worldwide in recent decades. The Food and Agriculture Organization of the United Nations (FAO) has reported that aquaculture produces over 50% of food fish consumed globally and is now the main source of increased fish supplies [4]. Aquaculture also has great potential for meeting customer demands such as improved safety and quality.

The leopard coral grouper, coral trout, *Plectropomus leopardus*, belongs to the family *Serranidae* and is distributed from the Western Pacific to East Africa and the Red Sea [5]. It is a commercially important species and trades at a high price. Increased demand has led to fish production via aquaculture including the development of aquaculture technology for leopard coral grouper. Feeding and metabolism are the greatest concerns in aquaculture.

The intestinal microbiota has been linked to a wide range of biological processes that benefit the host including nutritional conditions and the immune system [6–8]. Microbiota composition drastically changes in response to environmental and biological conditions [9–14]. The alternation of microbiota composition affects nutritional absorption and regulation of host energy balance [15–17] and interactions between host and gut microbiota caused by dietary conditions have been reported in many animals including fish [10, 18–21]. Moreover, the feeding and fasting cycle involves both host metabolism and gut microbiome composition and could lead to obesity and metabolic diseases [22]. The gut microbiota has also been shown to change according to the circadian rhythm because of the link with the host circadian rhythm [23]. Knowledge regarding the gut microbiota of teleostean fish has been gradually accumulating; however, information regarding dynamics and interaction with host fish remains limited and appears to differ according to condition and species.

In this study, we characterized the composition of the gut microbiome of the leopard coral grouper and investigated microbiome dynamics from fasting conditions to feeding conditions. The microbiota functional capacity was subsequently estimated and compared to the host transcriptome and metabolome data [24]. Our analysis suggests organization of the leopard coral grouper host metabolism and its microbiota metabolism.

## Materials and methods

### Fish and sampling

Eight-month-old leopard coral grouper weighing approximately 60 g were maintained in 60 kL tanks with a flow-through system at the Yaeyama Laboratory, Seikai National Fisheries Research Institute, Fisheries Research Agency. The facility was illuminated by sunlight with a natural photoperiod (11 L:13 D) during the winter. The fish were fasted for the first two days of the experiment and were then fed (Zeitgeber time (ZT) 2 and ZT10) to satiation for the next two experimental days. Feed (product name: Himezakura, HIGASHIMARU CO., LTD., Kagoshima, Japan) was purchased from a local supplier. The fish feed contains protein (>46.0%), fat (>10.0%), carbohydrates (<15.0%), fiber (<2.5%), calcium (>2.0%), and phosphorus (>1.0%). Gut contents were collected every 4 h, except during the night of Day 1 and Day 3. At each sampling point, three to twelve fish were sampled individually. The ethics statement is provided below. Seawater samples were collected from outside of the facility (costal seawater), the inlet, the rearing tank, and the outlet every 12 hours (ZT2 and ZT12). Skin mucus samples were collected from six fish at ZT10 of Day 2 (fasting) and Day 4 (feeding). Samples were collected and stored frozen until used for experiments.

### Ethics statement

All experiments were conducted in strict accordance with the principles and guidelines for the care and use of live fish and the guidelines for animal experimentation of the National Research Institute of Fisheries Science, Fisheries Research Agency. All experimental procedures were approved by the Animal Experimental Council (AEC/NRIFS) of the National Research Institute of Fisheries Science, Fisheries Research Agency. Fish were anesthetized with 2-phenoxyethanol (Wako, Osaka, Japan) and all efforts were made to minimize suffering.

### DNA extraction and DNA library construction

Gut content and skin mucus samples were collected and suspended in Tris-EDTA (TE) buffer with 10% sodium dodecyl sulfate (SDS). For the seawater samples, 15 mL of seawater, 33 mL of 70% ethanol, and 1.5 mL of 3 M sodium acetate were mixed and stored in a -20°C for 1 h. The seawater samples were centrifuged at 16,000 *g* for 20 min at 4°C. The obtained precipitates were washed with 70% ethanol and dried. DNA pellets were transferred into TE buffer containing 10% SDS. Samples in buffer were disrupted (10 min) using zirconium dioxide beads with an Automill machine (Tokken, Inc., Chiba, Japan). An equal volume of phenol-chloroform isoamyl alcohol (Wako, Osaka, Japan) was added followed by ethanol precipitation. The extracted DNA was used for CR amplification with universal bacterial primers 515F/806R targeting the V3–V4 regions of the 16S rRNA gene.

### Sequencing and data analysis

Amplified fragments were purified and sequenced using the Illumina MiSeq platform. Low quality reads were removed and clean reads were used to evaluate microbial community. The operational taxonomic units (OTUs) were calculated using the Qiime software [25]. Biodiversity was calculated with the Shannon indexes using the vegan package of R software. Hierarchical cluster analysis (HCA) was performed using CLC Genomics Workbench 8.1 (CLC bio, Aarhus, Denmark). Correlation analysis was performed and visualized with the Gephi software (https://gephi.org/). Microbiome data were analyzed by projection to latent structure-discriminant analysis (PLS-DA) using the R software, as previously described [21, 26, 27]. To investigate whether *Firmicutes* can serve as a marker for feeding and fasting, an artificial neural network self-organizing map (SOM) [28] was generated using the kohonen package of R software. Transcriptome and metabolome data were similarly analyzed by SOM. Representative transcripts and metabolites were selected based on our previous discriminant analysis results [24]. Microbiota functional capacity was estimated using the Piphillin software [29]. Piphillin evaluated the functional structure of the intestinal microbiome. Obtained KEGG IDs were converted to Clusters of Orthologous Groups (COG) IDs using the KEGG database (http://www.genome.jp/kegg/). The dynamics patterns of the putative functional genes were investigated. Eight metabolism categories, energy, carbohydrate, amino acid, lipid, nucleotide, inorganic ion, secondary metabolism, and coenzyme, were selected from COG category and normalized by Z-score in each category. HCA was performed on each functional gene using CLC Genomics Workbench 8.1. The HCA data were presented as Piphillin output results, which were not categorized. Functional genes correlated with the dynamics of the microbiota; *Firmicutes*, *Fusobacteria*, and *Proteobacteria* were selected by the R program. The number of correlated genes were calculated and presented by Z-score.

## Results

### Sequence and bacterial community composition

**Fig 1:**
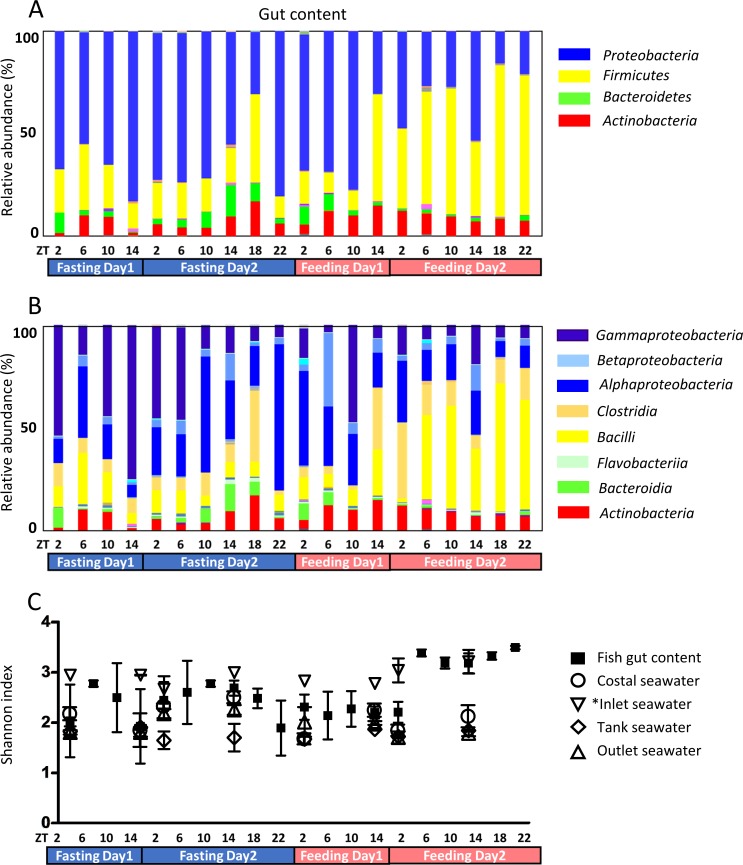
(A) Phylum level microbial taxonomic composition of gut contents. (B) Class level microbial taxonomic composition of gut contents. (C) Biodiversity of microbiota at each sampling point. Black squares designate the average and the bars indicate the standard error means. White circles denote costal seawater samples, white down-pointing triangles indicate inlet seawater samples, white diamonds signify rearing tank seawater samples, and white up-pointing triangles indicate outlet seawater samples. *Note that inlet seawater was subjected to UV sanitization; therefore, the number of microbiota was significantly low. The PCR cycles of inlet seawater was twice as many as other seawater samples. The replicate number of inlet seawater sequencing was one to two. NF, non-feeding; F, feeding; and ZT, Zeitgeber time.

Sequenced clean reads were obtained from each sample by MiSeq. Sequencing datasets have been submitted to the DDBJ Sequence Read Archive (DRA) under the accession number DRA006843. These reads were analyzed using the Qiime software and an OUT table was constructed. At the phylum level, four phyla, *Proteobacteria*, *Firmicutes*, *Actinobacteria*, and *Bacteroidetes*, accounted for >97% of the gut content. *Proteobacteria* was the dominant phylum (Fig 1A). *Firmicutes* gradually increased subsequent to feeding, while *Bacteroidetes* gradually decreased and became difficult to detect by the end of feeding. At the class level, *Alphaproteobacteria* and *Gammaproteobacteria* were the dominant *Proteobacteria*. *Clostridia* and *Bacilli* were the dominate *Firmicutes* classes, while *Flavobacteriia* and *Bacteroidia* were the major *Bacteroidetes* classes in gut content (Fig 1B). *Proteobacteria*, *Bacteroidetes*, *Firmicutes*, and *Actinobacteria* accounted for >99.7% of bacteria in seawater samples (S1 Fig). *Proteobacteria* was the dominant phylum in both gut content and seawater. At the class level, *Betaproteobacteria* and *Gammaproteobacteria* were the dominant *Proteobacteria*. *Clostridia* was the dominate *Firmicutes* class, while *Fravobacteriia* was the major *Bacteroidetes* class in seawater. The proportion of *Firmicutes* and *Actinobacteria* was greater in inlet seawater than in other seawater samples. In rearing tank seawater, the composition changed after feeding. The microbiota of skin mucus was also investigated (S2 Fig). *Proteobacteria* was the dominant class and the composition showed similar trends to the fasting gut microbiota. Gut microbiota biodiversity was higher after day 2 of feeding compared to the other experimental time points (Fig 1C). The biodiversity of seawater samples did not change during the experiment. The biodiversity of skin mucus did not change during the feeding period, but showed a slight decrease compared to the fasting period. However, the differences were not significant (S2 Fig).

### Clustering analysis and correlation analysis

**Fig 2:**
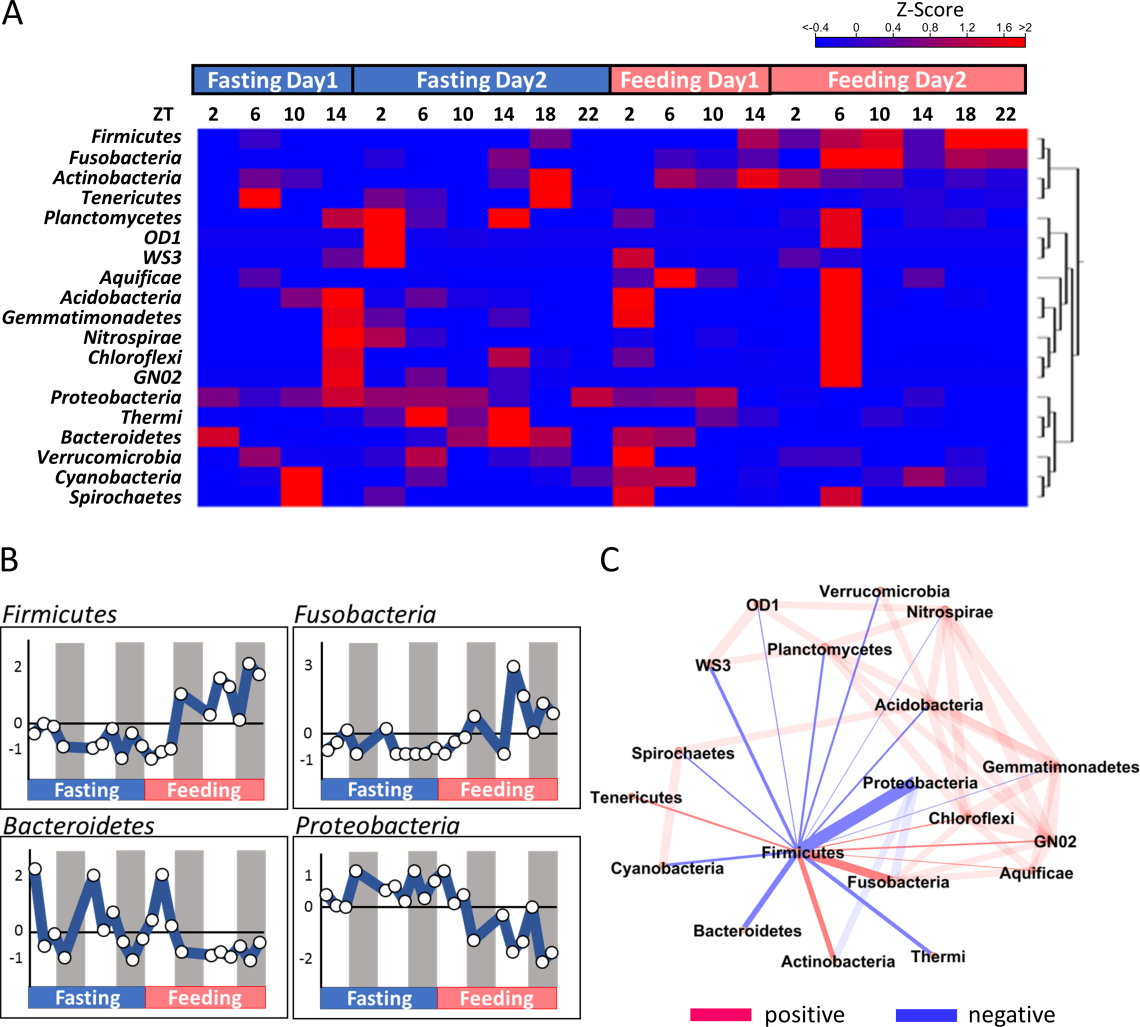
(A) Heatmap of microbiota abundance. Red, high abundance; blue, low abundance. The hierarchical cluster analysis tree is shown on the right. NF, non-feeding; F, feeding; and ZT, zeitgeber time. (B) Microbiota dynamics. White circles indicate the sampling points. (C) Network analysis of *Firmicutes*. Red denotes a positive correlation and blue designates a negative correlation. Thickness of the bar indicates the intensity of correlation; the thicker the bar the stronger the correlation.

HCA was performed and a heatmap was constructed to characterize the dynamics of bacterial community patterns (Fig 2A). Euclidean distance was utilized to measure the distance and clustering was conducted using the complete linkage analysis method. The HCA results showed that *Firmicutes* and *Proteobacteria* were located opposite to each other. The *Firmicutes* clade increased with feeding, while the *Proteobacteria* clade increased during fasting. The dynamics are shown in a graph (Fig 2B). Correlation analysis tests revealed that *Firmicutes* exhibited a positive correlation to *Fusobacteria* and a negative correlation with *Bacteroidetes* and *Proteobacteria* (Fig 2C).

### Discrimination analysis and clusterization by SOM

**Fig 3:**
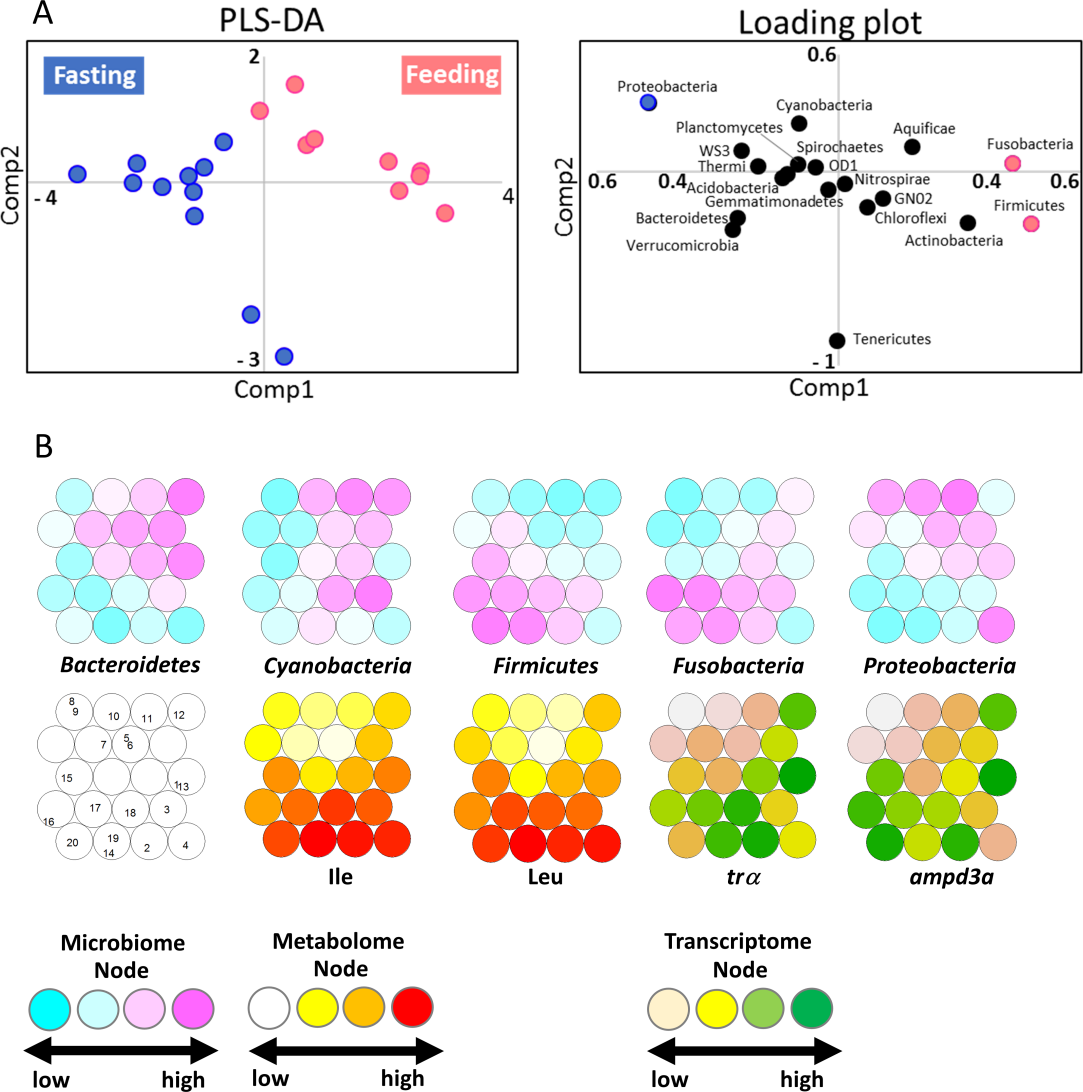
(A) Discrimination analysis by PLS-DA. Left, results described by the first and second component. Right, loading plot indicating the contributor expressed by reliability and intensity (B) SOM of data neural network analysis. The microbiota results are presented in blue and pink. Metabolome and transcriptome results are presented as red and greed gradation, respectively. The representative metabolites, isoleucine(Ile) and leucine (Leu) were chosen based on our previous data [24]. The representative genes, thyroid hormone receptor a (*trα*) and adenosine monophosphate deaminase 3a (*ampd3a*), were selected by OPLS-DA from our previous studies [24]. The class numbers of the samples are: 1, ZT2 of fasting Day1 (NF1-ZT2); 2, NF1-ZT6; 3, NF1-ZT10; 4, NF1-ZT14; 5, ZT2 of fasting Day2 (NF2-ZT2); 6, NF2-ZT6; 7, NF2-ZT10; 8, NF2-ZT14; 9, NF2-ZT18; 10, NF2-ZT22; 11, ZT2 of feeding Day1 (F1-ZT2); 12, F1-ZT6; 13, F1-ZT10; 14, F1-ZT14; 15, ZT2 of feeding Day2 (F2-ZT2); 16, F2-ZT6; 17, F2-ZT10; 18, F2-ZT14; 19, F2-ZT18; and 20, F2-ZT22.

The PLS-DA showed that the feeding group located in the negative side was distinguished from the fasting group located in the positive side. The PLS-DA score plot demonstrated that the metabolic profiles were also likely to cluster based on differences between the feeding and the fasting period (Fig 3A). The S-plot revealed that microbiota contributed to the discrimination profiles. The feeding group was characterized by *Firmicutes* and *Fusobacteria*, while the fasting group was characterized by *Bacteroidetes*. The SOM exhibited that fasting (group 1) was mainly located in the low node (blue), while feeding (group 2) was located in the high node (pink) (Fig 3B). Taken together, these findings indicate that *Firmicutes* could possibly constitute a marker of the feeding condition. In addition to microbiota, muscular transcript and metabolite data were also analyzed by SOM (Fig 3C and 3D). These data were derived from our previous study [24]. The mRNA of thyroid hormone receptor α (TRα) and leucine (Leu) were selected as representative markers by OPLS-DA [24]. The results showed that the fasting groups were located in low nodes and the feeding groups were located in the high node, indicating that TRα and Leu could be used as markers to distinguish between feeding and fasting conditions.

### Estimation of microbial function and comprehensive analysis

**Fig 4:**
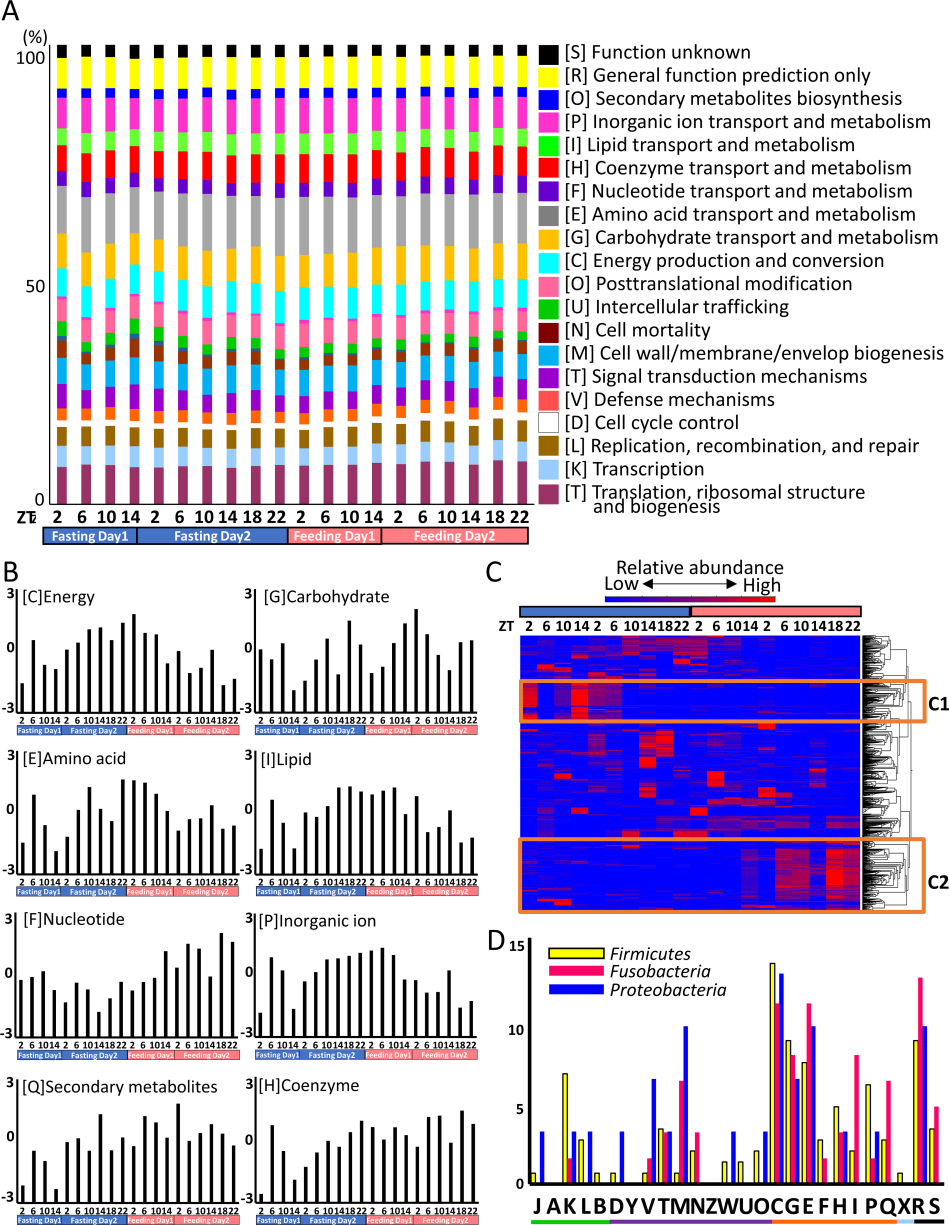
(A) Functional structure of intestinal microbiota exhibited by COG categories. (B) The dynamics pattern of the putative functional genes. Eight metabolism categories in COG classification; Y-axis, Z-normalization score. (C) Hierarchal clustering analysis of the presumptive functional genes. Highly expressed genes are in red and lowly expressed genes are in blue. The expression levels of genes in C1 were high in fasting and those in C2 were high in feeding. (D) Variation in the number of genes exhibiting dynamics similar to the microbiota. *Firmicutes*, yellow bars; *Fusobacteria*, pink bars; *Proteobacteria*, blue bars. Y-axis indicates the Z-normalization score. Capital letters indicate COG ID. The information storage and processing category (green bar) includes [J], Translation, ribosomal structure and biogenesis; [A], RNA processing and modification; [K], Transcription; [L], Replication, recombination and repair; and [B], Chromatin structure and dynamics. The cellular processes and signaling category (purple bar) includes [D], Cell cycle control, cell division, chromosome partitioning; [Y], Nuclear structure; [V], Defense mechanisms; [T], Signal transduction mechanisms; [M], Cell wall/membrane/envelope biogenesis; [N], Cell motility; [Z], Cytoskeleton; [W], Extracellular structures; [U], Intracellular trafficking, secretion, and vesicular transport; and [O], Posttranslational modification, protein turnover, chaperones. The metabolism category (orange bar) includes [C], Energy production and conversion; [G], Carbohydrate transport and metabolism; [E], Amino acid transport and metabolism; [F], Nucleotide transport and metabolism; [H], Coenzyme transport and metabolism; [I], Lipid transport and metabolism; [P], Inorganic ion transport and metabolism; and [Q], Secondary metabolites biosynthesis, transport and catabolism. The poorly characterized category (black bar) includes [R], General function prediction only; and [S], Function unknown. The light blue bar indicates [X], Mobilome: prophages, transposons.

Microbial function was predicted using Piphillin. The functional structure of the intestinal microbiome is shown in Fig 4A. No significant differences were observed for the COG categories between the samples. The dynamics of the functional profiling of the COG categories are exhibited in Fig 4B and S3 Fig. Fig 4B demonstrates the eight metabolism-related categories selected from Fig 4A; energy, carbohydrate, amino acid, lipid, nucleotide, inorganic ion, secondary metabolism, and coenzyme are presented as Z-normalization scores. Energy production [C], lipid metabolism [I], and inorganic metabolism [P] increased as starvation progressed. The hierarchical clustering analysis results are shown in Fig 4C. The heat map was constructed using the enrichment results of 7,531 KEGG functional orthologs (KO) and the data were calculated as Z-scores. Two characteristic clusters were identified. Cluster 1 (C1) exhibited the same dynamics pattern as *Proteobacteria* and cluster 2 (C2) exhibited the same dynamics pattern as *Firmicutes* and *Fusobacteria*. To further elucidate microbiota function, genes with correlated patterns were identified. Fig 4D shows the number of genes correlated with *Firmicutes*, *Fusobacteria*, and *Proteobacteria* (data are expressed as Z-scores) and S1 Table lists the genes correlated with these three phyla. Focusing on metabolism, only *Firmicutes* and *Fusobacteria* exhibited nucleotide transport and metabolism, lipid transport and metabolism, and secondary metabolite biosynthesis, transport, and catabolism in the feeding group. Gene details are listed in S1 Table; folate and purine nucleotide metabolism-related genes were identified among the nucleotide metabolism and transport genes.

## Discussion

The microbiota community was investigated under fasting and feeding conditions. *Proteobacteria* was the dominant phylum during fasting (Fig 1A), as well as in the seawater in the rearing tanks. Subsequent to feeding, *Firmicutes* became the dominant phylum. As fish were fed at ZT2 and ZT18 from day 3 and the dominant phylum had changed by ZT14 at day 3, it took approximately 12 hours for the dominant phyla to interchange. During fasting, *Gammaproteobacteria* were dominant, most likely originating from ingested seawater (Fig 1B and S1 Fig). Fish drink seawater to compensate for dehydration [30, 31]. In most cases, *Gammaproteobacteria* have been reported to be dominant in rearing tanks; the skin and gut of fish from both the sea and rearing tanks are rich in *Gammaproteobacteria* [19, 32–36]. In our study, *Gammaproteobacteria* was the most dominant class in the rearing tank seawater and skin mucus and the proportion of *Firmicutes* and *Actinobacteria* was higher in inlet seawater than in other seawater. As inlet seawater was subjected to UV sterilization, microbiota composition appears to have changed (Panel B in S1 Fig). Microbiota abundance was also significantly low due to sterilization. The microbiota in fish gut changed after feeding (ZT14 of Day3) and the composition of microbiota in rearing tank seawater changed simultaneously, suggesting that tank seawater is affected by fish feeding conditions. Skin mucus microbiota was also investigated (S2 Fig). The fasting gut content consists almost entirely of mucus; therefore, the skin mucus microbiota and the fasting gut microbiota were similar. Gut microbiota biodiversity was higher after day 2 of feeding compared to the other experimental time points (Fig 1C). In seawater samples, biodiversity did not change during the experiment, while skin mucus biodiversity slightly decreased during the fasting period. Feeding conditions might affect not only the digestive tract, but the entire body (S2 Fig). As microbiota composition changes in response to feeding and fasting conditions, it might also affect the host nutritional condition and it is hypothesized that the gut microbiota interacts with the host fish [10–13]. The alternation of microbiota composition affects nutritional absorption and regulation of host energy balance [15–17]. Although gut microbiota did not exhibit diurnal oscillation in our study [22, 23], our results show that fish microbiota composition changed within two days according to nutritional conditions.

Phylogenetic diversity changed according to feeding conditions (Fig 1C). The index was elevated after F4-ZT6 (Feeding Day 2, ZT 6), while the bacterial population increased after approximately 24 hours. A similar tendency of phylogenetic diversity under fasting conditions has been observed in tilapia [10]. Phylogenetic diversity has also been reported to change between wild and farmed fish [36]. Compared to the gut content, the phylogenetic diversity of seawater was low. Gut microbiota is thought to be complex in order to fulfill symbiosis functions.

HCA revealed three clusters: 1) high during fasting, 2) high during feeding, and 3) others (Fig 2A). *Proteobacteria* and *Bacteroidetes* belong to the fasting group, while *Firmicutes* and *Fusobacteria* belong to the feeding group. Subsequent correlation analysis revealed that *Firmicutes* and *Fusobacteria* exhibited a positive correlation, while *Firmicutes* and *Bacteroidetes* showed a negative correlation (Fig 2B and 2C). It has been widely established that *Firmicutes* increase under conditions of high fat feed intake and that *Firmicutes* and *Bacteroidetes* exhibit a negative correlation [37–39]. Although these reports examined mammals, fish have also exhibited a similar relationship between *Firmicutes* and *Bacteroidetes*. Our previous study showed the farmed fish store excess amounts of visceral fat [24]. This excess amount of fat is thought to originate from feed; therefore, the number of *Firmicutes* is predicted to increase.

The PLS-DA identified the contributor microbiota for feeding and fasting (Fig 3A). The feeding group was located in the negative side of T1, while the fasting group was in the positive side. Fasting conditions could be distinguished from feeding conditions. The key phyla were *Firmicutes* and *Fusobacteria* under feeding conditions and *Bacteroidetes* under fasting conditions. *Fusobacteria* are known to exist in the mammalian intestine and are sometimes found in the fish gut.

The microbiota, metabolome, and transcriptome data were integrated and analyzed by SOM (Fig 3B). SOM analysis using integrated data of several measurements can show multiple tendencies and evaluate variations in metabolic status. In addition, SOM maps dense and complex information into a two-dimension. This dimensionality reduction process requires unsupervised learning. The network exhibits the similarities and establishes increasingly complex relationship. This process enables the classification of datasets. Fasting and feeding status corresponded with the high and low nodes. In microbiota, *Firmicutes* and *Fusobacteria* showed similar patterns and the high nodes were mostly located in the feeding group. *Proteobacteria* were found to be inconsistent with *Firmicutes*. Muscular transcriptome and metabolome data was also subjected to SOM analysis. Genes and metabolites whose dynamics patterns corresponded to metabolic condition were selected from our pervious data [24]. The feeding groups tended to locate in the high nodes in the SOM map, while the fasting groups were located in the low nodes. However, the boundaries of feeding and fasting were not exactly the same, most likely because of different response times.

*Firmicutes* and *Fusobacteria* increased under feeding conditions and are predicted to interact with host metabolism. The functional prediction tools PICRUSt [40] and Tax4Fun [41] are widely used. Here, we utilized a new metagenomics inference tool, Piphillin [29], which improves existing tools and can be easily applied to any current genome database. The functional variability of the fish intestinal microbiome is shown in Fig 4A. The functional structure was stable during fasting and feeding. The abundance distribution of the fundamental functions is uniform in the human gut and the ocean [27, 39]. The detailed COG classification dynamics pattern demonstrated that energy production, lipid metabolism, and inorganic metabolism increased under fasting conditions (Fig 4B). During feeding conditions, the microbiota is predicted to utilize host gut nutrients and/or energy; however, it needs to generate energy independently under fasting conditions. Hierarchical clustering and heatmap analyses revealed the characteristic pattern of KEGG functional ortholog genes (Fig 4C). Two characteristic clusters were identified: Cluster 1 with a dynamics pattern identical to *Proteobacteria* and Cluster 2 identical to *Firmicutes* and *Fusobacteria*. Microbiota function was predicted by identifying correlated gene patterns (Fig 4D and S1 Table). Here, we focused on four phyla: *Firmicutes*, *Fusobacteria*, *Proteobacteria*, and *Bacteroidetes*. *Firmicutes* and *Fusobacteria* are predicted to be the contributors of feeding, which concluded in this study. *Proteobacteria* were the dominant species in fasting. *Bacteroidetes* also constitute a first contributor of fasting; however, the output numbers were too low to analyze. Thus, this study provides the results of three bacterial phyla (Fig 4D). Nucleotide transport and metabolism, lipid transport and metabolism, and secondary metabolite biosynthesis, transport, and catabolism were only detected in *Firmicutes* and *Fusobacteria*. Within the nucleotide transport and metabolism group, folate and purine nucleotide metabolism genes were detected (S1 Table). Genes involved in the nucleic acid component were also detected; bacterial DNA is predicted to be actively replicated [42, 43]. Among the lipid transport and metabolism and secondary metabolite biosynthesis, transport, and catabolism groups, fatty acid related genes were detected. Beta-oxidation is a pathway for degrading acyl-CoA into acetyl-CoA [44], which is finally integrated into the TCA cycle and contributes to energy production [45]. Metyl-malonyl CoA has also been shown to be converted into succinyl-CoA and integrated into the TCA cycle [46]. Thus, *Firmicutes* and *Fusobacteria* might promote fatty acid turnover in host fish.

Although the overall number of carbohydrate transport and metabolism genes detected did not differ significantly between feeding group and fasting group bacteria, the functions of the genes were totally different; sorbitol, trehalose, and maltose-related genes were detected only in feeding group bacteria. These three sugars were not detected in our previous metabolome data, suggesting that the amount of sugar was too low to detect or that these sugars do not circulate in the body [24]. However, gut mucosa homogenates have the ability to hydrolyze maltose and trehalose [47, 48]. Thus, *Firmicutes* and *Fusobacteria* existing in the gut mucosa could play a role in the hydrolysis of maltose and trehalose.

**Fig 5:**
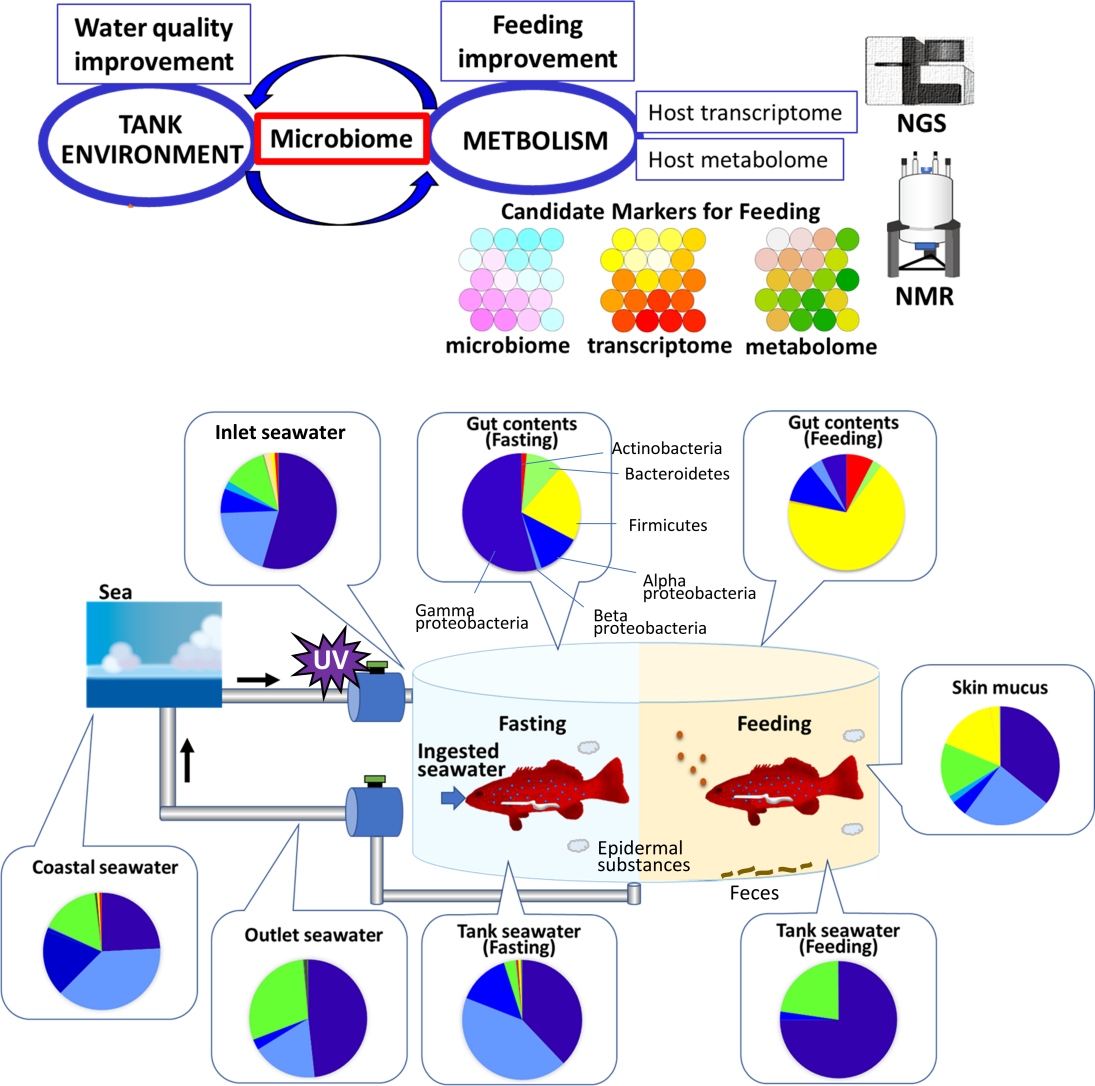
Overview of this study and proposed model for monitoring aquaculture environment and symbiotic metabolism upon feeding, as well as timing, via comprehensive analyses of microbiome, host metabolism, and host transcriptome.

We next investigated the dynamics of gut microbiota from fasting to feeding. Dominant bacteria during fasting are predicted to originate from ambient seawater. Following feeding, microbiota composition gradually changed and reached the feeding condition type 12 hours from the start of feeding. Multivariate analysis identified the following key bacteria: *Firmicutes* and *Fusobacteria* for feeding conditions and *Proteobacteria* for fasting conditions. *Gammaproteobacteria* was the dominant *Proteobacteria* class in ambient seawater, which is different from natural seawater. *Gammaproteobacteria* have been reported to be enriched in fish mucus, skin, and intestine; therefore, the *Gammaproteobacteria* in ambient seawater most likely originate from fish. In addition, our comparison of microbiome data with transcriptome and metabolome data by SOM analysis indicated microbial fluctuation following nutritional input is more significant than host metabolism [49–52]. Finally, comprehensive analysis of the microbiome and host metabolism could identify key factors for monitoring aquaculture environment and symbiotic metabolism upon feeding (Fig 5). Microbiota composition changed in response to feeding and fasting conditions and could affect nutritional control and energy balance. The feeding and fasting cycle involves host metabolism and is linked to obesity and metabolic diseases [22]. In this study, the composition of fish microbiota dramatically changed within two days of feeding and fasting cycles. As gut microbiota influence host fish metabolism, elucidating the relationship between gut microbiota and host fish might prove crucial for successful and efficient production of cultured fish. The fish microbiota can indicate the condition of the fish metabolism and immune systems. Environmental seawater directly affects fish conditions; therefore, monitoring microbiota in ambient seawater is also important for maintaining fish health. Controlling microbiota constitutes one approach for ensuring successful aquaculture.

## Supporting information

S1 Fig (A1) Phylum level and (A2) class level microbial taxonomic composition of coastal seawater. (B1) Phylum level and (B2) class level composition of inlet seawater. *Note that inlet seawater was sterilized and the number of microbiota was significantly low. The PCR cycles of inlet seawater was twice as many as other seawater samples. The replicate number of inlet seawater sequencing was one to two. (C1) Phylum level and (C2) class level composition of rearing tank seawater. (D1) Phylum level and (D2) class level composition of outlet seawater. ZT stands for the Zeitgeber time. ZT stands for the Zeitgeber time.
(TIF)

S2 Fig (A) Phylum level microbial taxonomic composition of skin mucus. (B) Class level microbial taxonomic composition of skin mucus. (C) Biodiversity of skin mucus microbiota.
(TIF)

S3 Fig The dynamics patterns of the putative functional genes obtained by Piphillin and categorized with COG. The data are expressed as Z-normalized score.
(TIF)

S1 Table Lists of the genes exhibited the correlation pattern as the dynamics of *Firmicutes*, *Fusobacteria* and *Proteobacteria*. (XLSX)
