# Supplementary figures and images for: Intestinal microbiota composition is altered according to nutritional biorhythms in the leopard coral grouper (Plectropomus leopardus)
Source: PLoS One. 2018 Jun 1;13(6):e0197256. doi: 10.1371/journal.pone.0197256 (PMC5983564; doi:10.1371/journal.pone.0197256)

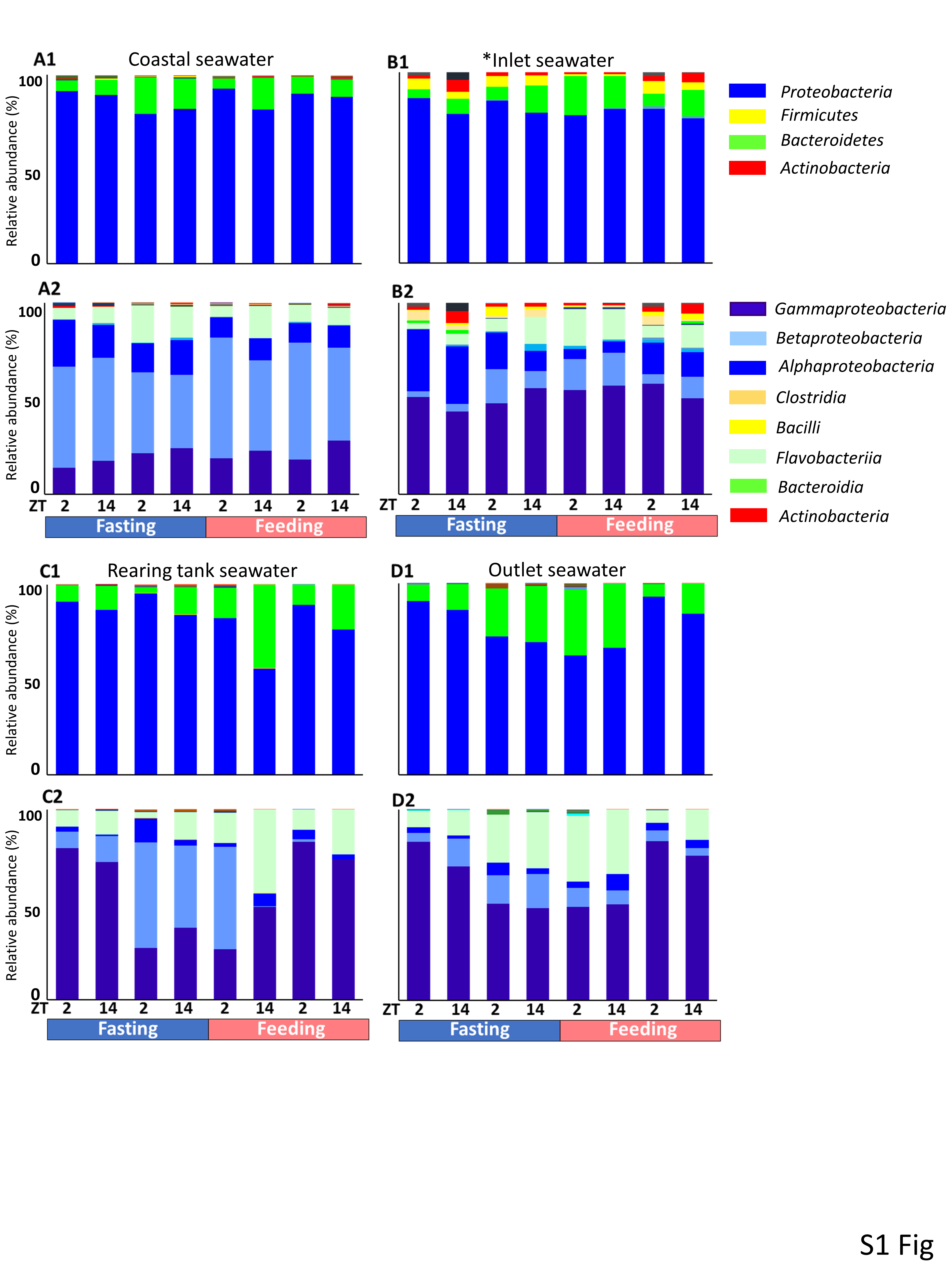

Supplement: S1 Fig — (A1) Phylum level and (A2) class level microbial taxonomic composition of coastal seawater. (B1) Phylum level and (B2) class level composition of inlet seawater. *Note that inlet seawater was sterilized and the number of microbiota was significantly low. The PCR cycles of inlet seawater was twice as many as other seawater samples. The replicate number of inlet seawater sequencing was one to two. (C1) Phylum level and (C2) class level composition of rearing tank seawater. (D1) Phylum level and (D2) class level composition of outlet seawater. ZT stands for the Zeitgeber time. ZT stands for the Zeitgeber time. (TIF) [file pone.0197256.s001.TIF]

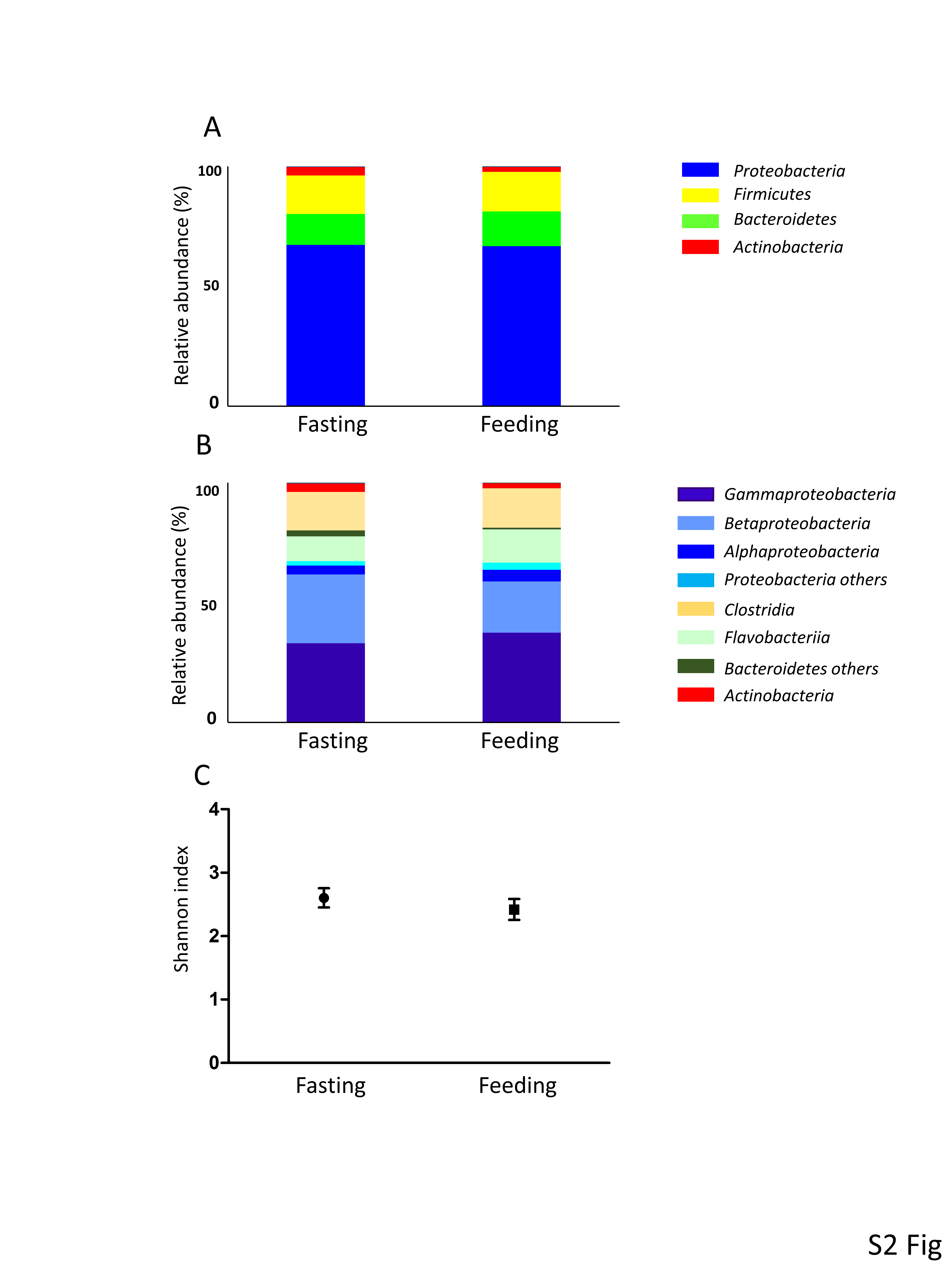

Supplement: S2 Fig — (A) Phylum level microbial taxonomic composition of skin mucus. (B) Class level microbial taxonomic composition of skin mucus. (C) Biodiversity of skin mucus microbiota. (TIF) [file pone.0197256.s002.TIF]

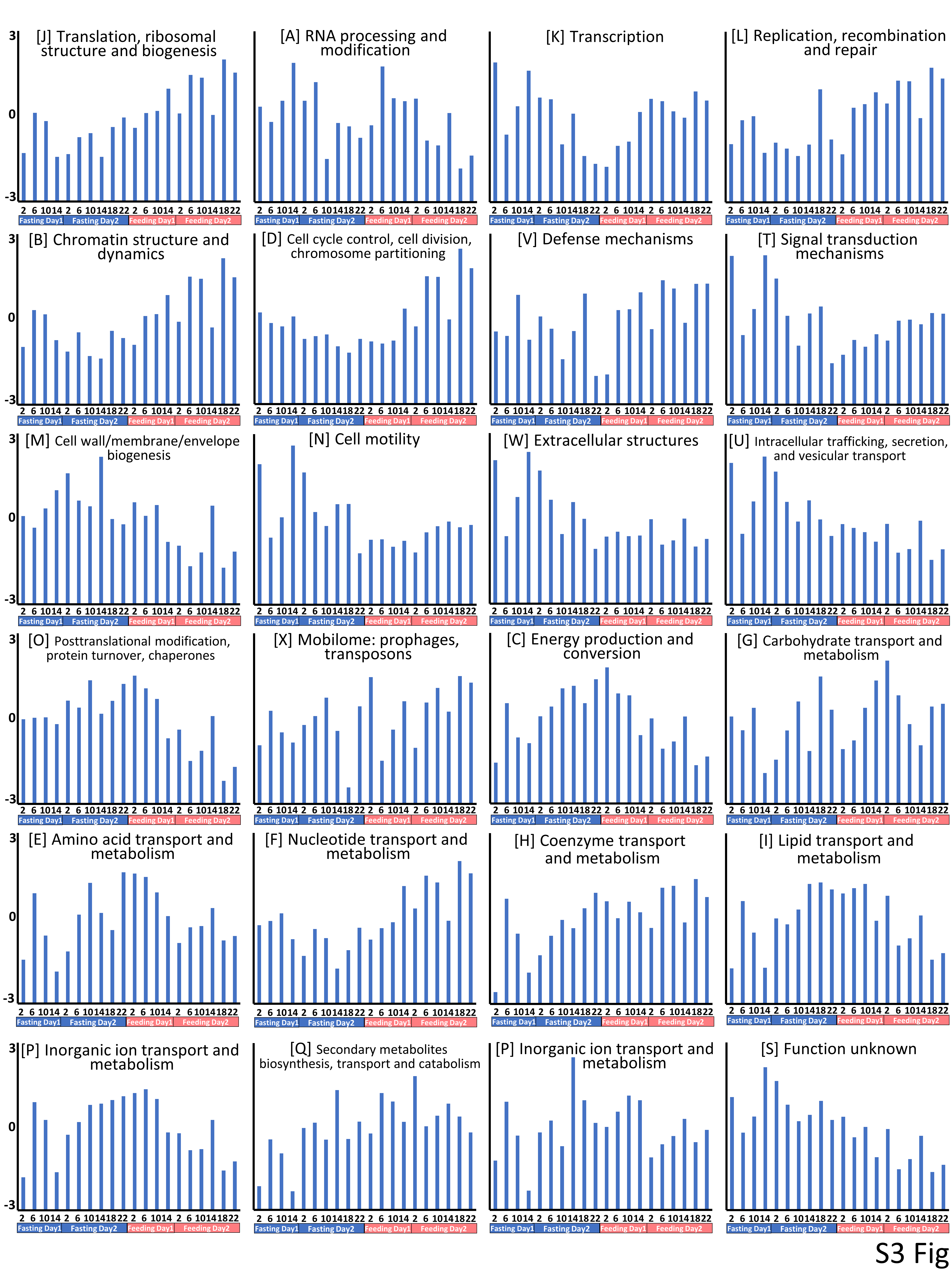

Supplement: S3 Fig — The data are expressed as Z-normalized score. (TIF) [file pone.0197256.s003.tif]
